# Repeated Lung Ultrasound versus Chest X-ray—Which One Predicts Better Clinical Outcome in COVID-19?

**DOI:** 10.3390/tomography9020056

**Published:** 2023-03-21

**Authors:** Jakob Spogis, Stefano Fusco, Florian Hagen, Sascha Kaufmann, Nisar Malek, Tatjana Hoffmann

**Affiliations:** 1 Department of Diagnostic and Interventional Radiology, Eberhard-Karls-University, Hoppe-Seyler-Str. 3, 72076 Tübingen, Germany; 2 Department of Internal Medicine, Eberhard-Karls-University, Hoppe-Seyler-Str. 3, 72076 Tübingen, Germany; 3 Department of Diagnostic and Interventional Radiology, Siloah St. Trudpert Klinikum, Wilferdinger Straße 67, 75179 Pforzheim, Germany

**Keywords:** lung ultrasound, X-ray, COVID-19, intensive care, critical care

## Abstract

The purpose of this study was to evaluate whether changes in repeated lung ultrasound (LUS) or chest X-ray (CXR) of coronavirus disease 2019 (COVID-19) patients can predict the development of severe disease and the need for treatment in the intensive care unit (ICU). In this prospective monocentric study, COVID-19 patients received standardized LUS and CXR at day 1, 3 and 5. Scores for changes in LUS (LUS score) and CXR (RALE and M-RALE) were calculated and compared. Intra-class correlation was calculated for two readers of CXR and ROC analysis to evaluate the best discriminator for the need for ICU treatment. A total of 30 patients were analyzed, 26 patients with follow-up LUS and CXR. Increase in M-RALE between baseline and follow-up 1 was significantly higher in patients with need for ICU treatment in the further hospital stay (*p* = 0.008). Both RALE and M-RALE significantly correlated with LUS score (r = 0.5, *p* < 0.0001). ROC curves with need for ICU treatment as separator were not significantly different for changes in M-RALE (AUC: 0.87) and LUS score (AUC: 0.79), both being good discriminators. ICC was moderate for RALE (0.56) and substantial for M-RALE (0.74). The present study demonstrates that both follow-up LUS and CXR are powerful tools to track the evolution of COVID-19, and can be used equally as predictors for the need for ICU treatment.

## 1. Introduction 

In December 2019, the novel coronavirus (SARS-CoV-2) broke out in Wuhan, China, with worldwide spread within a few months. The associated disease, coronavirus disease 2019 (COVID-19), is typically characterized by the symptoms of a viral pneumonia, which may evolve to respiratory failure. About 20% of hospitalized patients develop severe conditions with need for intensive care unit (ICU) treatment and potentially fatal outcome [1,2]. Chest imaging modalities, specifically chest X-ray (CXR) and computed tomography (CT), play an important role in the management of COVID-19 patients. In February 2019, the World Health Organization (WHO) published a rapid-advice guide for the use of chest imaging in suspected or confirmed cases [3]. For symptomatic patients, the WHO has recommended imaging as one element of the diagnostic workup, in addition to clinical and laboratory examinations to select patients requiring specific therapeutic management and to decide between hospitalization or patient discharge.

Assessment of lung injury is primarily focused on CT because of its high sensitivity to even minimal changes in lung parenchyma and its ability to effectively assess the disease’s progression [4,5]. There is broad evidence resulting from CT findings in COVID-19, most typically ground-glass opacities with or without consolidations in lung regions close to the visceral pleural surfaces and multifocal bilateral distribution [6]. Furthermore, CT is an excellent imaging modality to differentiate lung changes caused by COVID-19 from changes due to other conditions such as interstitial lung disease in systemic sclerosis, and to follow up with patients with long-term pulmonary impairments such as pulmonary fibrosis [7,8,9]. However, when performed on a regular basis and repeatedly, CT is a huge burden on the radiology department as well as on the human resources of the hospital [10].

In order to avoid these disadvantages, CXR is an easily available alternative which has been the most important first-line imaging modality for thoracic disease for many years and remains so even today [11,12]. Especially in acute respiratory distress syndrome (ARDS), CXR can monitor the course of the disease by visualizing the extent of pulmonary edema as a key feature of the disease [13,14]. In COVID-19, CXR mirrors the CT findings in the acute phase, such as bilateral lower zone consolidation with peripheral predominance. Although CXR can be used for diagnosis and monitoring of the disease, its sensitivity is lower than CT [15,16]. However, by quantifying the extent of pulmonary infiltrates, CXR is an effective radiological marker for predicting the in-hospital mortality and the need for invasive mechanical ventilation comparable to the values of chest CT [17].

There is broad experience in transthoracic lung ultrasound (LUS) in the evaluation of ARDS as it allows for a regional analysis of lung aeration [18,19,20]. Especially in point-of-care medicine and intensive care units, LUS plays an important role in lung imaging due to its portability, safety and repeatability. Furthermore, compared to other imaging modalities, LUS can produce real-time and dynamic images; additionally, non-specific, characteristic sonographic findings of COVID-19 lung involvement are irregular pleural line with multiple B-lines and subpleural consolidations with posterobasal predominance [21,22]. Multiple or confluent B-lines are the most common finding in COVID-19, in particular in ICU patients, where it may be present in nearly all cases [23]. Recent studies have confirmed that LUS has an accuracy similar to that of CT in detecting COVID-19-associated lung anomalies and that LUS could help in avoiding repeated CT scans [24,25]. Additionally, there is evidence supporting LUS being more sensitive in detecting COVID-19-associated lung changes compared to CXR, with a reported sensitivity of 92–96% compared to 46–69% in CXR [15,26,27,28].

We recently demonstrated in a prospective study with 30 COVID-19 patients that repeatedly performed LUS is able to indicate the development of severe disease [29]. We found increasing B-lines and pleural line irregularities as an indicator for ICU treatment in the further hospital stay. However, to the best of our knowledge, prospective studies comparing the predictability of CXR and LUS are still missing. This study aims, therefore, to assess the prognostic value of repeated CXR for need of ICU treatment in the same patient collective, and to compare it with repeated LUS.

## 2. Material and Methods

### 2.1. Study Design

This prospective, monocentric study was approved by the local Ethics Committee. Between March 2020 and September 2020, a total of 30 patients fulfilled the inclusion criteria (age > 18 years, positive SARS-CoV-2 PCR assay, in-patient care and ability to sign the informed consent) and were selected as participants.

### 2.2. Study Schedule

CXRs were performed at presentation in hospital and every second day consecutively. A maximum of three X-rays were analyzed (baseline, follow-up 1, follow-up 2). According to CXR, LUS was performed three times, the first within 48 hours of the first presentation in hospital and every second day consecutively. ICU treatment and death during the hospital stay were documented.

#### 2.2.1. Chest X-ray

CXRs were recorded with Agfa Retrofit (Agfa, Mortsel, Belgium) at bedside or in the emergency department in supine position in anterior-posterior projection. The X-rays were evaluated by two physicians, blinded to each other, with four and three years, respectively, of experience in clinical radiology. The Radiographic Assessment of Lung Edema (RALE) score was determined by the authors of a previous study [13]. Each radiograph was divided into four quadrants. Each quadrant was assigned a consolidation score from 0 to 4 quantifying the extent of pulmonary consolidation (0 = none; 1 = <25%; 2 = 25–50%; 3 = 50–75%; 4 = >75%) and a density score (1 = hazy; 2 = moderate; 3 = dense). The products of consolidation and density for each quadrant were summed for the final RALE score (range: 0–48). Additionally, the consolidation scores were added up neglecting the density score to calculate a modified RALE score (M-RALE, range: 0–16). Cardiothoracic ratio (CTR) was measured and presence of pleural effusion was noted.

#### 2.2.2. Lung Ultrasound

**Figure 1:**
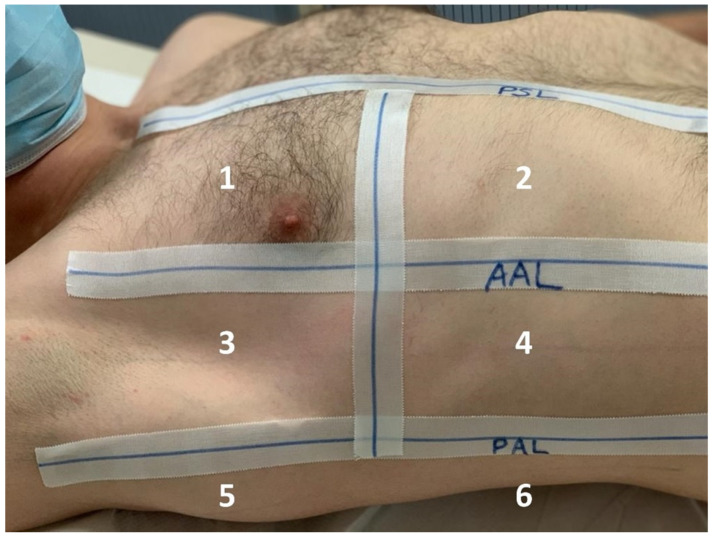
Lung areas for the right lung (labeled 1–6), PSL = parasternal line; AAL/PAL = anterior/posterior axillary line.

LUS studies were performed at the bedside by trained physicians with Philips Sparq (Philips Medical Systems, Hamburg, Germany). Convex C6-2 curved array with a 2–6 MHz frequency range and a 95 mm field of view and a linear L12-4 array transducer with a 4–12 MHz frequency range and a 34 mm field of view were placed longitudinally at each intercostal space of upper and lower parts of the anterior, lateral and posterior regions of the right chest wall (Figure 1). Videos of all 12 lung areas were recorded and evaluated in consensus by 2 physicians with 40 and 12 years of experience in clinical ultrasound, respectively, who were not the same physicians who reviewed the CXRs. Given that they are the most common finding in COVID-19, and to facilitate rapid and feasible examinations, our evaluation included the B-lines defined as comet-tail artefacts fanning out from the lung–wall interface and spreading up to the edge of the screen. Each lung area was assigned a semi-quantitative score (0 = none, 1 = moderate, 2 = heavy) adding up to the total LUS score (0–24). The LUS data collection and analysis are extensively described in [29].

### 2.3. Statistical Analysis

JMP 14.2.0 (SAS Institute Inc., Cary, NC, USA) and MedCalc 18.10 (MedCalc Software Ltd., Ostend, Belgium) were used for statistical analysis. Normally distributed data were given as mean ± standard deviation whereas non-normally distributed data were given as median and interquartile range (IQR). *T*-test and chi-square test were used to analyze RALE and M-RALE scores as well as their differences between the timepoints with regard to ICU treatment. The same tests were used to analyze the differences in CTR with regard to ICU treatment and to test for differences in the regional distribution of lung changes in CXR. Only after verification of Gaussian distribution of every parameter by the Shapiro–Wilk test, did we opt for a paired *t*-test. Linear regression was used to evaluate the correlation of RALE/M-RALE score and LUS score. Fisher’s exact test was used to compare increase/decrease in RALE and M-RALE scores as well as LUS scores between baseline and follow-up. To assess the reliability of RALE and M-RALE scores across independent reviewers, an average-measures intra-class correlation coefficient (ICC) was calculated. ICC values were interpreted as follows: 0–0.20 = poor agreement, 0.21–0.40 = fair agreement, 0.41–0.60 = moderate agreement, 0.61–0.80 = substantial agreement, 0.81–1 = (almost) perfect agreement.

In order to compare the predictive value of LUS and CXR, ROC curves were computed, with need for ICU treatment as binary separator. A *p*-value of less than 0.05 was considered statistically significant.

## 3. Results

**Figure 2:**
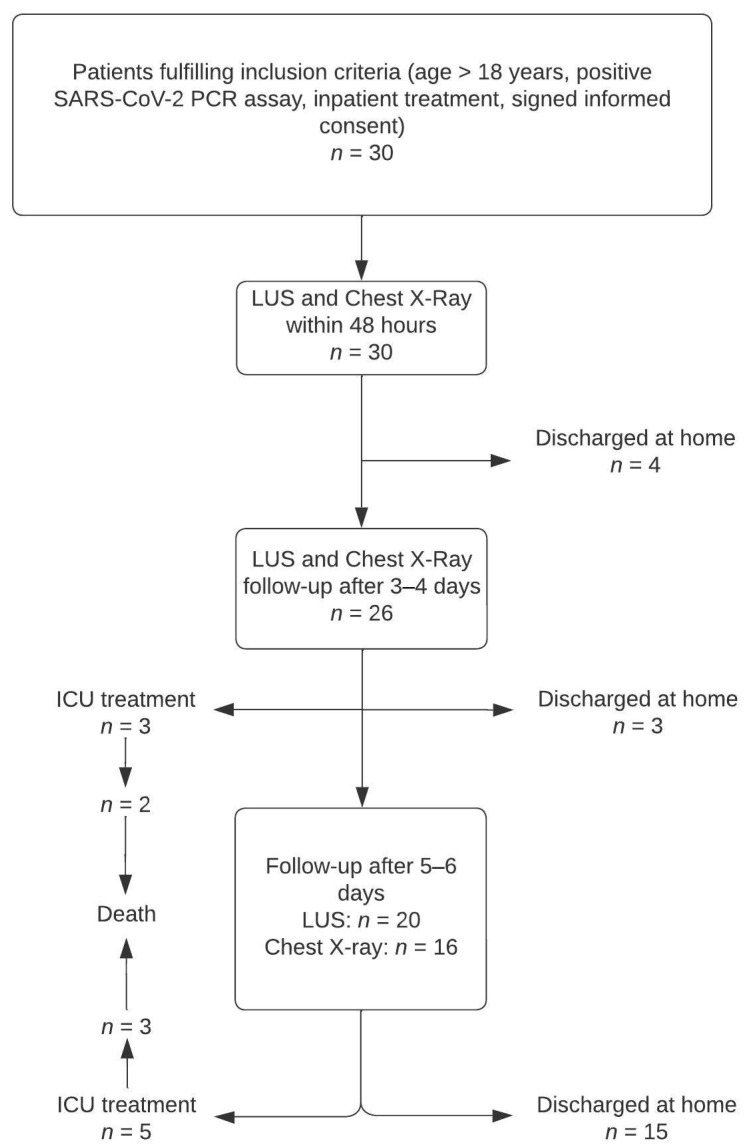
Study flow chart. Thirty patients fulfilled the inclusion criteria and were included in this prospective study. Twenty-six received at least one follow-up LUS and CXR.

Thirty patients were included in the study, of which eight (26.7%) had to be transferred to ICU and five (16.7%) died. Twenty-six (86.7%) patients received at least two CXRs and LUS; twenty (66.7%) received three LUS; sixteen (53.3%) received three CXRs. A total of 72 chest X-rays and 76 LUS were evaluated. The flowchart of the study is shown in Figure 2. Patient demographics are in Table 1. Median values were 8 (IQR: 4–13.5) for RALE, 5 (IQR: 2–7) for M-RALE, and 9.5 (IQR: 4.5–13) for the LUS score. Summary statistics for each timepoint are in Table 2. RALE score was significantly higher in the lower quadrants compared to the ipsilateral upper quadrants (right lung: mean difference = 1.13 (95% CI: 0.40–1.85), *p* = 0.001; left lung: mean difference = 2.21 (95% CI: 1.62–2.80), *p* < 0.001). No significant difference was found between the lower quadrants (mean difference=0 (95% CI: −0.66–0.66), *p* = 1).

ICCs for the evaluation of the CXR were moderate for RALE (ICC = 0.56, 95% CI: 0.38–0.70) and substantial for M-RALE (ICC = 0.74, 95% CI: 0.62–0.83). Mean CTR was 0.52 (standard deviation: 0.04) at baseline, 0.52 (standard deviation: 0.04) at follow-up 1, and 0.53 (standard deviation: 0.04) at follow-up 2. No significant difference between patients with and without need for ICU treatment in the further hospital stay was found (baseline: ICU mean = 0.51 (standard deviation: 0.04), non ICU mean = 0.53 (standard deviation: 0.04), *p* = 0.31; follow-up 1: ICU mean = 0.51 (standard deviation: 0.03), non ICU mean = 0.52 (standard deviation: 0.05), *p* = 0.64; follow-up 2: ICU mean = 0.53 (standard deviation: 0.03), non ICU mean = 0.54 (standard deviation: 0.04), *p* = 0.44). Small pleural effusions were detected in three patients (one on the left, one on the right, and one on both sides), with one effusion progressing at follow-up 1 and 2 while the others remained stable.

### 3.1. Association of RALE and Need for ICU Treatment

At baseline, no statistically significant difference between patients with and without need for ICU treatment in the further hospital stay could be detected (RALE: ICU median = 8.5 (IQR: 4.5–14), non ICU median = 4.5 (IQR: 2–9.25), *p* = 0.19; M-RALE: ICU median = 4.5 (IQR: 3.25–7), non ICU median = 3 (IQR: 1.75–5.75), *p* = 0.41), whereas at follow-up 1 and 2, M-RALE was significantly higher in patients with further need for ICU treatment (follow-up 1: ICU mean = 7 (standard deviation: 2.07), non ICU mean = 4.3 (standard deviation: 2.68), *p* = 0.01; follow-up 2: ICU mean = 7.43 (standard deviation: 2.07), non ICU mean = 3.89 (standard deviation: 1.97), *p* = 0.02). Accordingly, the difference in M-RALE score between baseline and follow-up 1 was significantly higher in the ICU group (*p* = 0.008, difference: 2.3, 95% CI: 0.69–4.03), whereas there was no significant difference between follow-up 1 and follow-up 2 (*p* = 0.19, difference: 1.48, 95% CI: −0.86–3.81). At each follow-up, the RALE score did not show significant differences between the two groups (follow-up 1: ICU median = 12 (IQR: 8.25–17), non ICU median = 5.5 (IQR: 3.75–13.25), *p* = 0.07; follow-up 2: ICU mean = 13.7 (standard deviation: 8.52), non ICU mean = 8.44 (standard deviation: 5.94), *p* = 0.19). Additionally, there was no significant difference between baseline and follow-up 1 (*p* = 0.07, difference: 4.13, 95% CI: −0.56–8.81) or follow-up 1 and follow-up 2 (*p* = 0.49, difference: 1.95, 95% CI: −4.1–8.0) for RALE. No patient with stable or decreasing RALE or M-RALE score on follow-up 1 compared to baseline needed ICU treatment in the later hospital stay, whereas 8/15 (53.3%) of patients with increase at follow-up 1 were transferred to ICU later on (*p* = 0.007).

### 3.2. Comparison of CXR with LUS 

**Figure 3:**
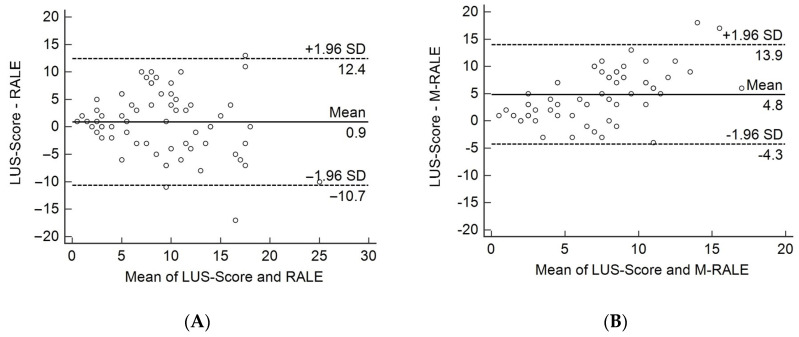
Bland–Altman plots for (**A**) LUS score vs. RALE and (**B**) LUS score vs. M-RALE. Both RALE and M-RALE showed a moderate, significant correlation with LUS score, with only a few outliers in the Bland–Altman plots.

**Figure 4:**
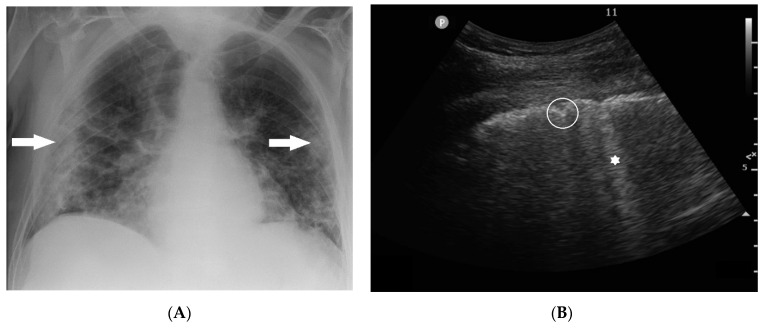
(**A**) Chest X-ray and (**B**) lung ultrasound of an 81-year-old patient with consolidations at the periphery of both lungs (arrow) in CXR and corresponding loss of aeration with well-defined B-lines (asterisk) and pleural line irregularities (circle) in LUS at presentation in hospital. LUS was performed with a convex C6-2 probe with a 2–6 MHz frequency range.

Both RALE and M-RALE showed a moderate, significant correlation with LUS score (r = 0.52; *p* < 0.0001 and r = 0.50; *p* < 0.0001, respectively, Figure 3 and Figure 4). Sixteen of nineteen (84.2%) patients with a decreasing or stable LUS score at follow-up 1 compared to baseline were discharged without ICU treatment, whereas five/seven (71.4%) with an increasing LUS score were transferred to ICU (*p* = 0.014).

**Figure 5:**
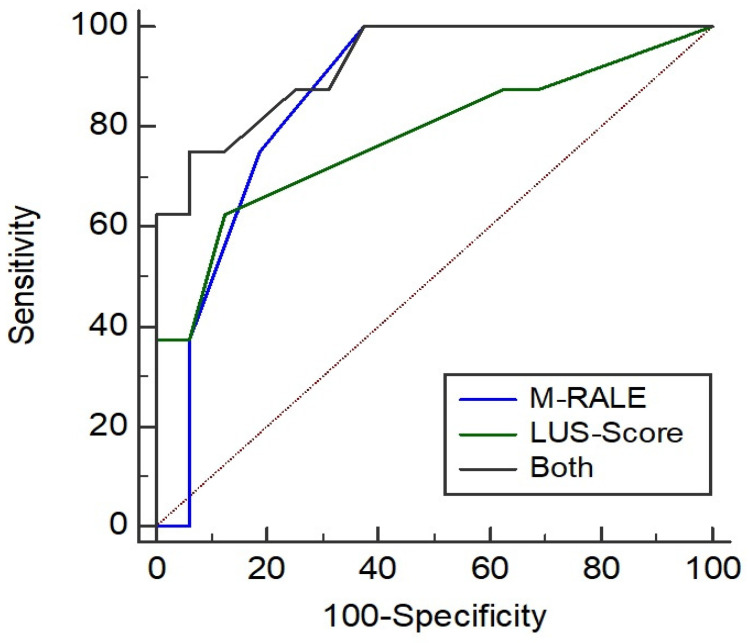
ROC analysis for differences between baseline and follow-up 1: M-RALE (blue line, AUC: 0.87, best cut-off point: 0, sensitivity: 100%, specificity, 61.1%), LUS score (green line, AUC: 0.79, best cut-off point: 0, sensitivity: 62.5%, specificity: 88.9%) and both added up weighted for the maximum values (black line, AUC: 0.92, best cut-off point: 0.15, sensitivity: 75.0%, specificity: 93.7%) showed a good prediction for the need of ICU treatment; no significant difference was found.

ROC curves with need for ICU treatment as binary separator for differences in M-RALE and LUS score between baseline and follow-up 1, and both values added up weighted for the maximum values are shown in Figure 5. The AUC of the M-RALE difference (0.87; 95% CI: 0.75–0.97), the LUS score difference (0.79; 95% CI: 0.56–0.92) and both added up (0.92; 95% CI: 0.74–0.99) were not significantly different (*p* = 0.06–0.37).

## 4. Discussion

In the present study, 26 out of 30 patients underwent at least 2 CXRs and LUS. Lung consolidations in our cohort were predominantly in the lower parts of the lungs, which is in line with previous findings in COVID-19 [15]. At baseline, no significant difference in CXR scores could be detected between patients with a need for ICU treatment in the further course vs. patients who were discharged at home, whereas at first follow-up after 3–4 days a significant difference in M-RALE could be detected. Changes on days 5–6 did not gain further information with regard to the need for ICU treatment. With reference to the CXR scores, M-RALE showed better results than RALE. That means that the quantitative extent of lung changes seems to be more predictive for ICU treatment than the combination with the density of these changes. This is most likely due to the fact that ground-glass-like opacities are predominant in the early phase of the disease and consolidations most often occur later on [30]. The patients in our study were examined in the early phase of the disease when the extent of lung involvement seems to be the important clinical factor. Pleural effusions could possibly interfere with lung changes due to COVID-19 in projection radiography, leading to alterations in the RALE score. However, in our cohort only three patients had detectable pleural effusions. Therefore, it is unlikely that pleural effusions had a significant impact on the lung scoring. Forty percent of our patients had a history of a cardiac disease such as chronic heart failure or coronary artery disease. The cardiothoracic ratio (CTR) > 0.5 is a crude marker for diagnosis of heart failure [31]. To obtain reliable values, CTR should only be measured in posterior–anterior CXRs. However, in our study, CXRs were acquired in anterior–posterior projection, leading to generally higher values. Despite this, we found no significant difference between the two groups, indicating that this was not a confounder.

When comparing CXRs and LUS, changes in CXRs appeared to be more sensitive for predicting ICU treatment in the further course than LUS. This is because no patient with stable or decreasing RALE or M-RALE had to be transferred to ICU. On the other hand, LUS was more specific than CXRs because more patients with an increasing LUS score had to be transferred to ICU than patients with an increasing RALE or M-RALE. Although LUS showed only a moderate, significant correlation with RALE and M-RALE, the AUC for changes between baseline and follow-up 1 showed good results for both LUS score and M-RALE. This indicates that both modalities are good discriminators and not significantly different. A combined score resulted in a small, non-significant improvement of the AUC, indicating that both modalities reflect similar lung changes and can be used equally. This is interesting because one possible drawback of LUS is the inability to visualize changes in deeper lung areas. However, in our study for COVID-19, looking beyond the surface of the lung using CXRs did not provide any additional benefit to track the evolution of the disease.

COVID-19 is a highly contagious disease with a significant influence on public health. In individual patients, the clinical course is highly dynamic and may progress to acute respiratory distress syndrome [32]. Due to infection-control concerns related to patient transport and its widespread availability, portable CXR is likely the most commonly utilized modality for the evaluation of suspected thoracic diseases and follow-up of lung abnormalities [11,12]. On the other hand, in recent years LUS has gained increasing interest in the field of intensive care and especially in ARDS because of its ability to assess lung aeration changes and lung edema as well as the regional distribution of these changes [11,18,33]. As COVID-19 pneumonia progresses in the distal regions of the lung, we already know that it is well suited to a surface imaging technique such as LUS [34]. Its role in evaluating COVID-19 is well-documented and we could recently show that it is suitable for tracking the disease’s progression [22,29,35,36]. The aim of this study was to compare the role of repeated CXRs and LUS in assessing and predicting the course of COVID-19, particularly the need for ICU treatment. Among the advantages of LUS are that it is a portable, non-radiative, repeatable and routinely, easily cleaned bedside modality to detect a range of typical pulmonary patterns in COVID-19 patients. On the other hand, LUS requires more time and personnel resources to be performed and interpreted compared to CXRs. Additional advantages of CXRs include its lack of examiner dependency, ease of comparing to previous examinations and ability to examine the entire lung in one image. However, in our cohort the objectivity of CXRs may be limited, as intra-class correlation was moderate for RALE and M-RALE. As there were only a few confounding factors, such as pleural effusion, the moderate intra-class correlation observed in CXRs may be due to the supine position of the patient and the anterior–posterior projection, which can lead to a less standardized exposure and projection.

CT is widely used to rapidly assess the severity of COVID-19 and remains an important imaging tool in clinical routine due to its ability to visualize even discrete lung changes and differentiate them from other lung diseases [6,8,30]. This said, due to infection-control concerns, lack of availability and limited resources, CT is often reserved for severe or inconclusive cases. Chest digital tomosynthesis (DTS) could be an interesting alternative to CXR, as it can overcome the disadvantage of overlapping clutter and preserve the advantage of lower radiation dose compared to standard chest CT [37,38,39]. However, it is not frequently used and cannot be performed at the bedside. DTS could therefore play an emerging role in follow-up of chronic changes following COVID-19, where a detailed visualization of the lung parenchyma and the reticular and fibrotic changes is needed [37].

This study has some limitations. First, it was conducted in a single-center design combined with a small size sample. This limited the power to explore the association between findings in CXR and LUS. Nevertheless, even in this cohort we could demonstrate that both modalities are equally powerful discriminators for the further course of COVID-19 in the early phase. Second, this study lacks CT scans as the gold standard for COVID-19 pneumonia. Due to this limitation, we could not determine which of the two modalities was closer to one approaching the “ground truth”. However, as said before, both performed equally well. The main strength of our study is that we had a prospective design, with repeated CXRs and LUS ruling out selection bias. Additionally, all examinations were evaluated by experienced physicians. However, larger studies are necessary to strengthen the role of LUS and CXRs in monitoring COVID-19.

## 5. Conclusions

In conclusion, we could demonstrate that repeatedly performed CXRs and LUS are both powerful imaging modalities for the monitoring of COVID-19. Changes at day 3 can be a reliable indicator of the need for ICU treatment in the further hospital stay. Both modalities have their advantages and can be used complementary or together, depending on the local conditions. Although CXR is a standard technique with broad experience and the advantage of generating objective images, LUS may be a powerful, easily learnable tool for clinicians in the emergency ward or the ICU.

## Figures and Tables

**Table 1:** Patient demographics and clinical baseline characteristics.

Age, Mean (*n*, SD)	70 (13.3)
Sex, male (*n*, %)	17 (57%)
Transfer to ICU (*n*, %)	8 (27%)
In-hospital mortality (*n*, %)	5 (16%)
Prior history of heart disease (*n*, %)	12 (40%)
Prior history of lung disease (*n*, %)	11 (37%)
Prior history of immunosuppressant therapy (*n*, %)	3 (10%)
Prior history of cancer (*n*, %)	7 (23%)
Initial oxygen supplementation (*n*, %)	16 (53%)
Fever (≥38.3 °C) (*n*, %)	8 (27%)
Dyspnea (*n*, %)	14 (47%)
Tachypnea (≥20/min) (*n*, %)	24 (80%)
Oxygen saturation (<95%) (*n*, %)	17 (57%)
Early warning score ≥8 (*n*, %)	14 (47%)

**Table 2:** Summary statistics of RALE, M-RALE and LUS scores at baseline, follow-up 1 and 2. Data are shown as median and interquartile range.

	Baseline Median (IQR)	Follow-Up 1 Median (IQR)	Follow-Up 2 Median (IQR)
RALE	5.5 (2–13)	10 (4–14)	10 (6–14)
M-RALE	3.5 (2–7)	5 (2–8)	5 (3–7)
LUS score	8 (4–12)	10 (6–13)	10 (5–12.5)

## Data Availability

Data are contained in the Supplementary Materials. The data presented in this study are available in Table S1.

## Supplementary Materials

The following supporting information can be downloaded at: https://www.mdpi.com/article/10.3390/tomography9020056/s1, Table S1: Data supporting the reported results.
